# Mast Cell Degranulation and Adenosine Release:Acupoint Specificity for Effect of Electroacupuncture on Pituitrin-Induced Acute Heart Bradycardia in Rabbits

**DOI:** 10.1155/2020/1348914

**Published:** 2020-10-07

**Authors:** Xuezhi Wang, Meng Huang, Hongwei Yang, Di Zhang, Wei Yao, Ying Xia, Guanghong Ding

**Affiliations:** ^1^Shanghai Key Laboratory of Acupuncture Mechanism and Acupoint Function, Department of Aeronautics and Astronautics, Fudan University, Shanghai 200433, China; ^2^Shanghai Research Center for Acupuncture & Meridian, Shanghai 201203, China

## Abstract

Acupuncture is a medical modality based on the theory of traditional Chinese medicine, and its effect is relatively dependent on acupoint specificity. However, there is little knowledge on acupoint specificity versus acupuncture outcomes because of the deficiency of rigorous investigation on this topic, which has impeded the growing legitimacy of acupuncture in the mainstream of medicine as an evidence-based therapy. Therefore, it is of utmost importance to clarify this critical issue. The present study aims to verify the phenomenon of acupoint specificity in acupuncture-induced cardiovascular regulation and explore the biological mechanism by measuring mast cells' degranulation and adenosine release. This study was conducted to explore the specificity of acupoints in an acute bradycardia rabbit model. After electroacupuncture (EA) stimulation at PC6, PC control (con) 1, PC con 2, LU7, LI11, and nonacupoint, only the PC6 group showed a significant improvement in relative heart rate as compared to that of the model group. There was no significant difference between the relative heart rate of other EA groups and that of the model group. Historical results also showed that the ratio of degranulated mast cells in PC6 was significantly higher than other acupoints and control points. From the results of high-performance liquid chromatography (HPLC), a transient elevation of adenosine concentration during EA was only observed on acupoints and control points (*P* < 0.05) along the pericardium meridian. The EA-induced adjustment on acute bradycardia exhibits a relative specificity of acupoints, which may be related to mast cell degranulation and adenosine release in local acupoint areas. Increased degranulation of mast cells and augmentation of adenosine release during EA may be the mechanisms for PC6 having significantly better acupuncture effects than other acupoints and nonacupoints.

## 1. Introduction

This study attempts to observe the acupoint specificity and investigate the biological mechanism behind it from the events of degranulation of mast cells and adenosine release caused by acupuncture. In the past two decades, based on high-quality basic and clinical research, acupuncture research has made considerable progress, and the efficacy of acupuncture has been recognized worldwide [1–9]. However, the acupuncture researchers have always avoided discussing acupoint specificity, the core proposition of acupuncture theory, hindering the growth of legitimacy in the mainstream of medicine as an evidence-based therapy [10]. There are very few clinical and basic studies discussing acupoint specificity, and the research conclusions are controversial [11–15]. A recent randomized clinical trial on chronic stable angina found that receiving acupuncture on the disease-affected meridian can significantly reduce angina attacks compared to receiving acupuncture on the acupoints on the nonaffected meridian, receiving sham acupuncture, or receiving no acupuncture [16]. The results of two clinical studies in patients with migraine indicate that the brain glucose metabolism caused by acupuncture at acupoints is pertinent and targeted, and acupuncture at different acupoints can lead to different glucose metabolism levels in pain-related brain regions [17, 18]. However, the application of acupuncture on nonacupoints induced disordered and randomized brain glucose metabolism. A study relying on the lipopolysaccharide-induced endotoxemia mouse model found that EA at ST36 drives the vagal-adrenal axis producing anti-inflammatory effects depending on NPY^+^ adrenal chromaffin cells. EA at ST25 activates NPY^+^ splenic noradrenergic neurons through the spinal-sympathetic axis [19], suggesting that neuronal activation induced by EA at different acupoints is specific. Nevertheless, an individual patient meta-analysis of 17,922 patients with chronic pain in randomized controlled trials showed that there is no evidence that any characteristic of acupuncture modifies the acupuncture analgesic effect, including the style of acupuncture, the number or placement of needles, the number, frequency or duration of sessions, patient-practitioner interactions, and the experience of acupuncturists [20]. Given these controversies, acupuncture specificity and its underlying biological basis must be further elucidated as soon as possible.

## 2. Materials and Methods

### 2.1. Animals

One of the selection principles for nonacupoint is to close to the target point and maintain a certain distance from distinction. Therefore, the area of the medial forelimb of the experimental animal selected in this experiment should not be too small. According to this standard, we excluded rats and mice, which are commonly used experimental animals. The distances between PC6 and control points on rabbit medial forelimb are moderate. Besides, the rabbit's ear is quite large, and the blood vessels are manifested, which is convenient for repeated intravenous injection. So, the rabbit is suitable as an experimental animal model of the long-term acute bradycardia model.

Male New Zealand rabbits (2.0 ± 0.2 kg) from Shanghai Shengwang Experimental Animals Ltd. (permit number SCXK(SH)-2012-0007) were housed in cages in a temperature-controlled environment (22–25°C) with a 12/12-hour light/dark cycle. Food and water were freely available. All animals were handled with care to prevent infection and to minimize stress. Animal experiments were performed between 9 am and 5 pm. For each experimental group, animals were chosen randomly. All animals were housed for at least 2 days before experimentation.

### 2.2. Anesthesia

After rabbits were prepared, a 20% urethane solution (7.5 ml/kg) was injected into the right ear vein using a venous indwelling needle, and injection duration was controlled to be approximately 60 s. When using urethane anesthesia, the rabbit anesthesia time is more than 2 h, which is enough to support the complete experimental process, and the anesthesia effect is stable. Compared with other anesthetic drugs, the animal mortality rate is low, and the impact on heart rate and intraventricular pressure is small.

### 2.3. Long-Term Acute Myocardial Ischemia Model

Male New Zealand rabbits weighing 2.0 ± 0.2 kg were used in this study. Anesthesia (7.5 ml/kg) was injected into the ear vein with 20% urethane. Then, the animals were placed on the surgical table in a supine position, and Electrocardiograph (ECG) signals were collected. After resting for 20 min, pituitrin (3 units/ml, 2 units/kg) was injected into the ear vein followed by a low concentration of pituitrin (36 units/L) being administered at a rate of 1 ml/min using a microinjection pump until the end of the experiment. A 30% reduction in heart rate from the baseline 2 min after modeling was considered a sign of successful modeling.

### 2.4. ECG Recording

After anesthesia, animals were placed on the surgical table, and ECG signals were recorded using a two-lead subcutaneous connection. ECG signals were introduced into the Powerlab multichannel physiological recorder (Powerlab 16/30 AD Instruments) via the ECG module (Powerlab Dual Bio Amp ML135 AD Instruments).

### 2.5. Groups

**Figure 1:**
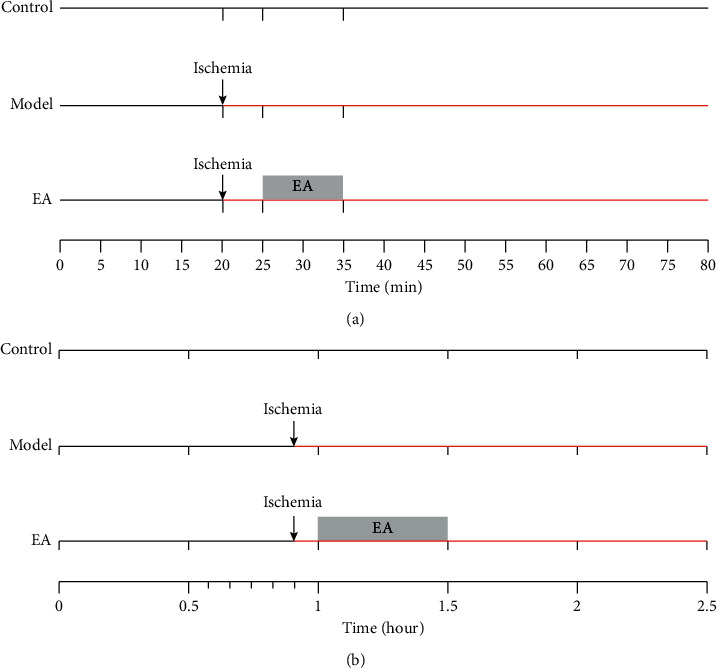
Flow chart of experiments. (a) Flow chat of applying acupuncture in the experimental bradycardia model. (b) Flow chart of microdialysis experiments.

48 rabbits were randomly divided into 8 groups. The blank group (Control) animals were only used to record data and underwent no other procedures. The model group (Model) was placed onto the experimental bench for 20 min to induce a long-term acute myocardial ischemia model. No EA was applied after modeling. The Neiguan group (PC6) was placed onto the experimental bench for 20 min to prepare a long-term acute myocardial ischemia model. After modeling was completed, EA was applied at the PC6 for 5 min. The operation of the pericardium meridian control 1 point group (PC con 1), the pericardium meridian control 2 point group (PC con 2), the Lieque group (LU7), the Quchi group (LI11), and the nonacupoint group (Nonacupoint) were the same as that of the PC6 group. The only difference was the EA stimulation position among these EA groups (the PC6 group, the PC con 1 group, the PC con 2 group, the LU7 group, the LI11 group, and the nonacupoint group). The complete experimental procedure is shown in Figure 1(a). Two rabbits were unsuccessful in modeling, and another rabbit had interference when recording ECG data. These three rabbits were not included in statistical analyses.

Other 48 rabbits were randomly divided into 8 groups. Rabbits in the blank control group (Control) were placed onto the experimental operating table, and only tissue fluid under PC6 was collected. Samples were collected every 30 min, and a total of 5 samples were collected over 2.5 h with no other procedures. Rabbits in the model group were placed onto the surgical table, and tissue fluid under PC6 was collected. Samples were collected every 30 min, and a total of 5 samples were collected. The total length of the experiment was 2.5 h, and the acute myocardial ischemia model lasted 55 min. After modeling, EA treatment was not performed. Rabbits in the Neiguan microdialysis EA group (PC6) were placed on the surgical table, and tissue fluid under PC6 was collected. Samples were collected every 30 min, and a total of 5 samples were collected. The total length of the experiment was 2.5 h, and the acute myocardial ischemia model lasted 55 min. EA was applied at PC6 for 30 min starting at 5 min after the modeling. Procedures for pericardium meridian control 1 point microdialysis EA (PC con 1), pericardium meridian control 2 point microdialysis EA (PC con 2), Lieque microdialysis EA (LU7), Quchi microdialysis EA (LI11), and nonacupoint microdialysis EA (Nonacupoint) groups were the same as those in the Neiguan microdialysis EA group (PC6); only the positions of EA stimulation and microdialysis fluid collection were different. The complete experimental procedure is shown in Figure 1(b). Two rabbits were not included in the statistical analyses due to partial loss.

### 2.6. Acupoints and Control Acupoints

**Figure 2:**
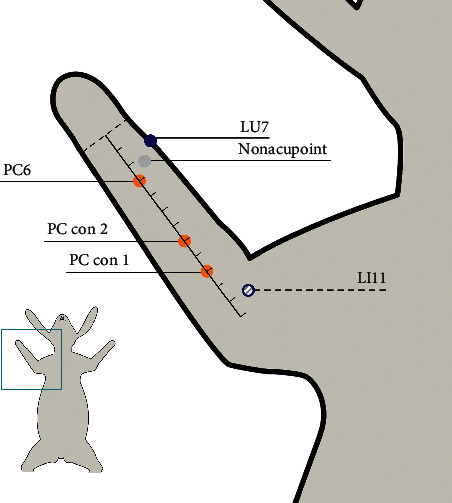
Schematic of acupoints and other points on the body surface of a rabbit. The lower left panel shows a schematic of the rabbit in a supine position. The green frame was enlarged to show a detailed forearm partial view on the right side. The dashed line on the wrist is a reference line of the transverse crease of the wrist. According to the rabbit common acupoint location standard in Experimental Acupuncture (29), PC6 is designated as the point at 1/4 of the anterior forearm length above the transverse crease of the wrist between the tendons of the palmaris longus and flexor carpi radialis. On the palmar side of the forearm, between the radius and ulna, the 9 cun position above the transverse crease of the wrist is the PC con 1, and the 7 cun position is the PC con 2. Since PC6 and all control points are on the pericardium meridian, they are uniformly marked with orange solid dots. LU7 is located on the lateral side of the forearm, on the upper edge of the styloid process of the radius, 1.5 cun above the transverse crease of the wrist, between the brachioradialis muscle and the tendon of the long abductor muscle of thumb, represented by a dark blue solid dot. Quchi is designated as the point at the depression on the outer side of the elbow joint. In particular, since its position cannot be observed in the supine position, it is indicated by a dark blue dotted hollow dot. The nonacupoint position is taken from the midpoint of the connection between PC6 and LU7. Since its location is not on any meridian, it is indicated by a solid gray dot.

Figure 2 visually shows the location of acupuncture points and other points on the rabbit's body surface.

PC6 is clinically used to regulate cardiac function [21, 22]. Traditional Chinese medicine has long known that “symptoms of heart and chest should be treated by stimulating PC6.” Therefore, we used PC6 as the target point in this study. The anatomical position of PC6 is between the tendons of the palmaris longus and flexor carpi radialis [23], which is the flexor digitorum sublimis, and the deep layer is the flexor digitorum profundus. That location includes the median artery and vein of the forearm, and the deep layer includes the artery and vein between the bones on the palmar side of the forearm. The medial forearm cutaneous nerve is at this location, and the deep layer is the median nerve palmar branch, with the deepest layer housing the forearm volar interosseous nerve. Clinically, the PC6 acupoint is designated as the point at the middle of the palmar side of the forearm, 2 *cun* above the transverse crease of the wrist, between the tendons of the palmaris longus and flexor carpi radialis [23]. In this experiment, the positioning method for defining PC6 was from the rabbit common acupoint location standard of Experimental Acupuncture [24]. PC6 is designated as the point 1/4 of the anterior forearm length above the transverse crease of the wrist between the ulna and the radius in this experiment.

For PC con 1 and PC con 2, the selection principle of the position of control points on the pericardium meridian includes (1) avoiding acupoints on the same meridian as much as possible and (2) getting as close as possible to PC6 while trying to avoid the difference in acupuncture effect caused by the different segments. As shown in Figure 2, the acupoints are relatively dense at the distal end of the forearm, and there is no acupoint between 5 *cun* above the transverse crease of the wrist and cubital crease. Therefore, the position 9 *cun* above the transverse crease of the wrist is taken as the position of PC con 1 and 7 *cun* as PC con 2 as a compromise. According to the comparative anatomical method, PC con 1 is indicated as the point 7/12 of the anterior forearm length above the transverse crease of the wrist between the ulna and the radius, and PC con 2 is indicated as the point 3/4 of that length.

For LU7 and LI11, the selection criteria of control acupoints are (1) avoiding acupoints on the pericardium meridian, (2) not including myocardial ischemic disease regions, and (3) getting as close as possible to PC6 at the same level. Therefore, we selected LU7 as a control acupoint on another meridian. LU7 is located on the lateral side of the forearm, on the upper side of the styloid process of the radius, 1.5 *cun* above the transverse crease of the wrist, between the brachioradialis muscle and the tendon of the long abductor muscle of the thumb [23]. LU7 is close to PC6, but there is a difference in the indications between them, which is a good control point in this experiment. Since LU7 is not a common acupoint in animal experiments, there is little authoritative literature for defining the location of LU7. Therefore, according to the comparative anatomical method, LU7 is located on the lateral side of the forearm, on the upper edge of the styloid process of the radius, 1/8 of the anterior forearm length above the transverse crease of the wrist, between the brachioradialis muscle and the tendon of the long abductor muscle of the thumb. The anatomical position of LI11 is located at the beginning of the extensor carpi radialis longus, where the radial artery and cephalic vein are distributed, as well as the radial nerve and the dorsal cutaneous nerve of the forearm. Clinically, LI11 is located at the lateral end of the transverse cubital crease with the elbow flexed, the midpoint between the connection of LU5 and lateral epicondyle of the humerus [23]. In this experiment, the method of defining LI11 was based on the rabbit common acupoint location standard of Experimental Acupuncture [24]. LI11 is designated as the point at the depression on the outer side of the elbow joint.

For nonacupoint positions, the principles of nonacupoint selection are (1) being close to PC6 while leaving a certain distance to clearly distinguish it from PC6, (2) trying to stay far away from other acupoints, and (3) avoiding the pericardium meridian and other meridians. According to these three principles of selecting nonacupoints, the midpoint of the connection between PC6 and LU7 was taken as the nonacupoint region in this research.

### 2.7. EA Stimulation

According to the size of the selected experimental animal, the depth of the lesion, and the location of the acupoint, a 0.25 × 13 mm sterile acupuncture needle was selected as the experimental needle. All acupoints and control acupoints, except LU7, were subjected to perpendicular insertion. The oblique insertion method was used separately at LU7. The depth of needle penetration for all acupoints and control points, which is 7 mm, was referenced to the standard of the rabbit common acupoint location of Experimental Acupuncture [24]. EA stimulation intensity was uniformly 0.4 mA—the minimum current intensity that can be induced by stimulating PC6 to cause slight jitter of the forearm. The output frequency and waveform were 2/100 Hz alternately dense waves with a duration of 10 min. In the microdialysis experiment, the duration of EA was 30 min, while other parameters remain unchanged.

### 2.8. Microdialysis

This experiment used a linear probe (MD-2005, Basi, USA) with a 5 mm semipermeable membrane. The dialysis solution was Ringer's solution, and 1 mM/L adenosine deaminase inhibitor (EHNA hydrochloride E114, Sigma, USA) was added to Ringer's solution. The dialysis power was supplied by a syringe pump (NE-1000, New Era Pump Systems, USA) with a flow rate of 2 *µ*l/min. From the beginning of the dialysis process, one sample was collected every 30 min for 2.5 h. A total of 5 samples were collected, each with a volume of 60 *µ*l. The samples were immediately placed on ice and then stored at −80°C until HPLC analysis.

### 2.9. HPLC Detection

An Agilent 1260 HPLC analyzer was used as an analytical device. Chromatographic separation was performed by a reversed-phase column EC-C18. For the measurement of microdialysis fluid, we used a mobile phase containing 215 mM KH_2_PO_4_, 2.3 mM tetrabutylammonium bisulfate, and 3.2% acetonitrile (pH 6.2) with a flow rate of 0.3 ml/min. The adenosine standard was separately checked every 5 samples, and the dialysis sample was compared to the standard according to the peak area to calculate actual concentration values.

### 2.10. Histological Examination

To obtain the number and degranulation ratio of mast cells in acupoints and control acupoints, rabbits were exposed to inhalation carbon dioxide and sacrificed after experiments were completed. Taking the PC6, LU7, nonacupoint, PC con 1, PC con 2, and LI11 of the right forelimb of the rabbit as the center, skin and skeletal muscle tissue samples of 5 × 5 × 5 mm^3^ around those points were carefully dissected. All tissue samples were immediately soaked in Carnoy's solution for 20 h. After dehydration and waxing, they were trimmed into small pieces of approximately 3 × 3 × 3 mm^3^ and embedded in paraffin. The sectioning direction was perpendicular to the skin surface, and one sample was taken every 0.15 mm to prepare a paraffin section with a thickness of 5 *µ*m and subjected to complex dyeing of toluidine blue and safranine T (0.5 mol/L HCL 5 min, 0.5 mg/ml toluidine blue (PH = 0.5) 45 min, 0.5 mol/L HCl 30 s, 0.25 mg/ml safranine T 30 s) and then sealed.

### 2.11. Data Analysis and Statistics

ECG was recorded immediately after the completion of the operation, and recording was completed at the end of the experiment. In this experiment, the heart rate was recorded using Lab Chart Pro 7.0 software. The raw data sampling rate was 20 k/s, and 3 Hz high-pass digital filtering was performed during data recording. The original ECG data were extracted and averaged in units of 60 s before statistical analysis. Heart rate was normalized based on the heart rate 5 min before modeling. The relative heart rate of the x-minute is calculated as R_HR_(x) = A_HR_(x)/(∑_*i*=15_^19^A_HR_(*x*)/5), where A_HR_(x) represents the x-minute absolute heart rate value. All data were statistically analyzed by F-test, and based on the results of the F-test, a single-tailed *T*-test was performed for data comparison among groups. All heart rate data processing after extraction from Lab Chart Pro 7.0 was performed in Excel.

Under the 40 × 10 X light microscope, the diameter of mast cells was approximately 10∼20 *µ*m. The shape of an intact mast cell is elliptical or fusiform with a clear edge, and the color is a dark brownish purple. Degranulated mast cells are incomplete and elliptical or fusiform with blurred edges or irregularly broken, and their color is light brown. The number of intact mast cells and degranulated mast cells in the field of view of 230 *µ*m × 310 *µ*m was counted under a 40 × 10 X light microscope. Three sections were taken from each animal, and 3 fields were observed for each section. The total number of mast cells and the number of degranulated mast cells in each field of view were recorded separately. The ratio of degranulated mast cells = the number of degranulated mast cells/the total number of mast cells × 100%. The average single visual field degranulation ratio was taken as the tissue degranulation rate at the acupoint or control point. All data were statistically analyzed by F-test, and based on the results of the F-test, a single-tailed *T*-test was performed for data comparison among groups. All data were processed in Excel.

Five samples were obtained after completion of the microdialysis experiment with a total duration of 2.5 h. To eliminate the influence of microdialysis needle insertion, the concentration of the first sample was not counted. The concentration of the sample collected during the second period (30∼60 min) was used as the base concentration. Then, the concentration values of the samples collected at subsequent periods were normalized. The x-minute relative concentration value was calculated as *R*_D_(x) = *A*_D_(x)/(∑_*i*=2_^5^A_D_(*x*)/A_D_(2)). *A*_D_(x) represents the absolute concentration of adenosine collected from period *x*. The intragroup comparison data were analyzed using a single-tailed paired *T*-test. All data were statistically analyzed by F-test, and based on the results of the *F*-test, a single-tailed *T*-test was performed for data comparison among groups. All data were processed using Excel.

## 3. Results

### 3.1. EA Adjustment of Heart Rate

**Figure 3:**
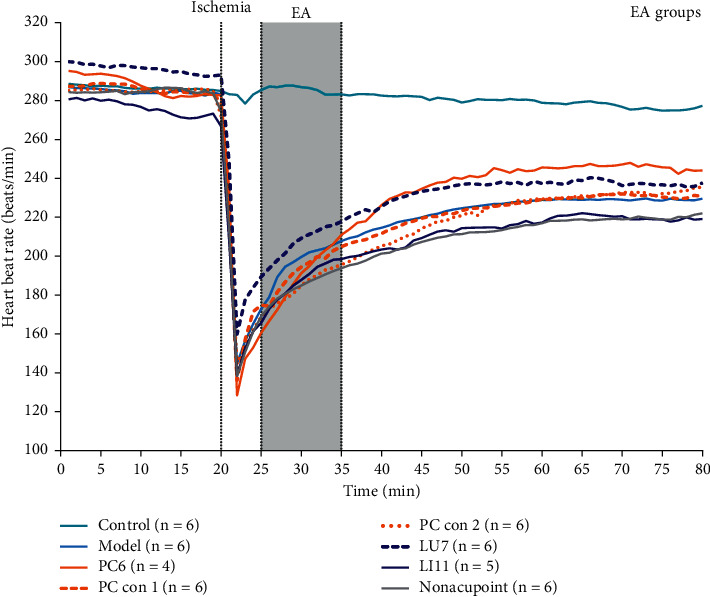
Differences in heart rate among different groups. The heart rate of different groups is detected by the Powerlab multichannel physiological recorder (Powerlab 16/30 AD INSTRUMENTS) (*n* = 4∼6). The abscissa shows observation time in min. The ordinate is the heart rate in units of beats/min. The green solid line represents the control group (Control), the light blue solid line represents the model group (Model), the orange solid line represents the Neiguan group (PC6), the orange dashed line represents pericardium meridian control point 1 (PC con 1), the orange dotted line represents pericardium meridian control point 2 (PC con 2), the dark blue dashed line represents the Lieque group (LU7), the dark blue solid line represents the Quchi group (LI11), and the gray solid line represents the nonacupoint group (Nonacupoint).

To determine the existence of acupoint specificity, we tested the acupoint specificity on the pericardium meridian in acute bradycardic rabbits. As shown in Figure 3, pituitrin (2 Unit/kg) was injected into the animals after recording 20 min of heart rate, which caused a sharp drop in heart rate in rabbits. Heart rate dropped to the lowest point at approximately 2 min after the injection. Receiving 10-minute EA treatment starting at 5 min after modeling, heart rate recovery in each group showed distinct trends based on characteristics of acupoints or control points.

**Figure 4:**
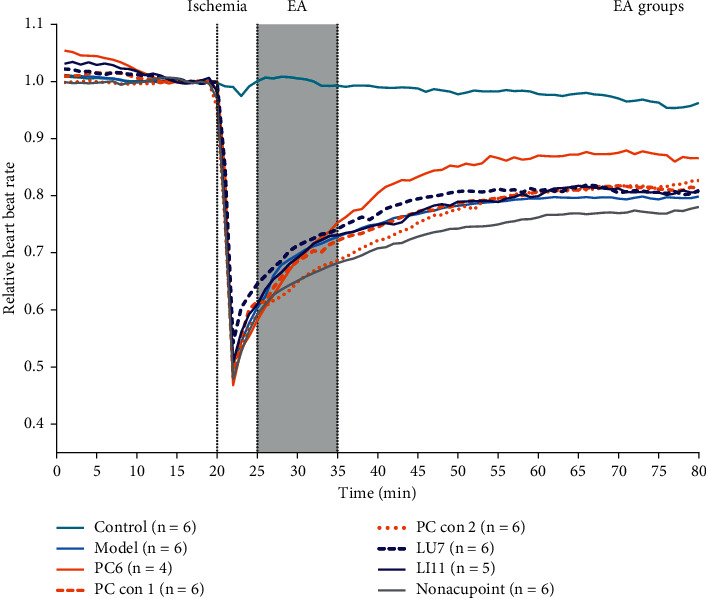
Relative heart rate among different groups. The relative heart rate of different groups is calculated against the basic heartbeat (*n* = 4∼6). Relative results are shown in Figure 2. The abscissa shows observation time in min. The ordinate is the relative heart rate in units of 1. The green solid line represents the control group (Control), the light blue solid line represents the model group (Model), the orange solid line represents the Neiguan group (PC6), the orange dashed line represents pericardium meridian control point 1 (PC con 1), the orange dotted line represents pericardium meridian control point 2 (PC con 2), the dark blue dashed line represents the Lieque group (LU7), the dark blue solid line represents the Quchi group (LI11), and the gray solid line represents the nonacupoint group (Nonacupoint).

Because of variations in basic heart rate (*P* < 0.05), we normalized the heart rate data against the basic heartbeat for better comparison among various groups (*P* > 0.05). The average 5 min basic heart rate before modeling, 15–20 min in Figure 3, was taken as the baseline to recalculate the data. Relative results are shown in Figure 4.

**Figure 5:**
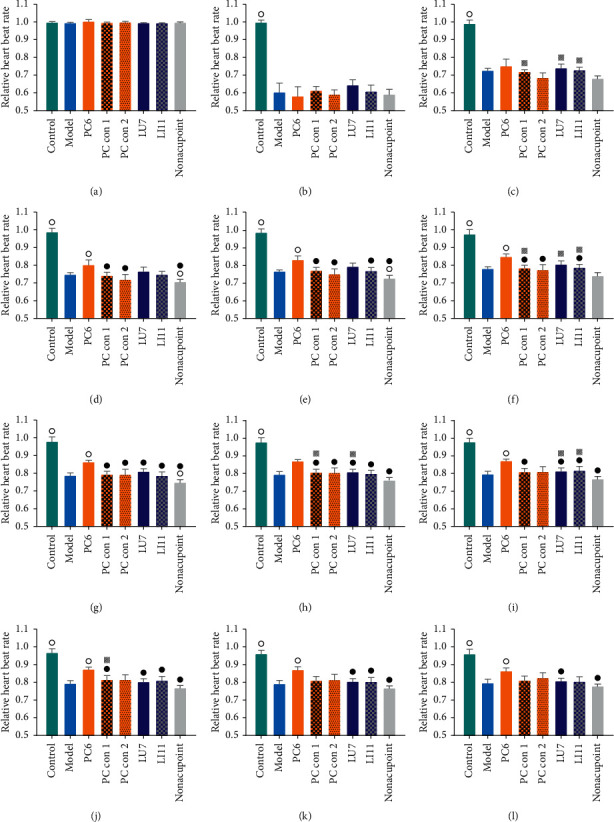
Relative heart rate index among different groups. A comparison of the relative heart rate index was made among different groups (*n* = 4∼6). ○ indicates a significant difference compared to the model group (*P* < 0.05); ● indicates a significant difference compared to the PC6 group (*P* < 0.05); ■ indicates a significant difference compared to the PC con 1 group (*P* < 0.05); □ indicates a significant difference compared to the PC con 2 group (*P* < 0.05); 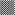 indicates a significant difference compared to nonacupoint group (*P* < 0.05); 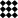 indicates a significant difference compared to LU7 group (*P* < 0.05). (a) 19 min. (b) 25 min. (c) 35 min. (d) 40 min. (e) 45 min. (f) 50 min. (g) 55 min. (h) 60 min. (i) 65 min. (j) 70 min. (k) 75 min. (l) 80 min.

The comparison of the relative heart rate is shown in Figure 5. Compared with the model group, the control group exhibited a significant difference from the beginning of modeling to the end of the experiment. The PC6 group demonstrated a significant advantage from 40 min until the end of the experiment (*P* < 0.01 from 45 to 75 min). The nonacupoint group exhibited a significant disadvantage at 35–45 min and 55 min. Compared to the PC6 group, the nonacupoint group demonstrated a significant disadvantage from 35 min to the end of the experiment. The LU7 group has a significant disadvantage from 55 min until the end of the experiment. The PC con 1 group exhibited a significant disadvantage from 40 min to 70 min, but there was no significant difference from 75 min to the end of the experiment. The PC con 2 group showed a significant disadvantage from 40 to 60 min. The LI11 group exhibited a significant disadvantage during 45–75 min. Compared to the nonacupoint group, the LU7 group had a significant advantage during 35–65 min. The PC con 1 group displayed a significant disadvantage at 35 min, 45–65 min, and 70 min, while the PC con 2 group demonstrated no significant disadvantage. The LI11 group had a significant advantage at 35–40 min, 50 min, and 65 min.

The above results suggest that acupuncture at PC6 has a comparative advantage, better than other stimulated points. The relative heart rate recovery index in the PC6 group is not only superior to the model group but also better than other EA groups. Similarly, the acupuncture effects of PC con 1, LU7, and LI11 were significantly better than the nonacupoint group.

### 3.2. Effect of EA on Local Mast Cells in Acupoints

**Figure 6:**
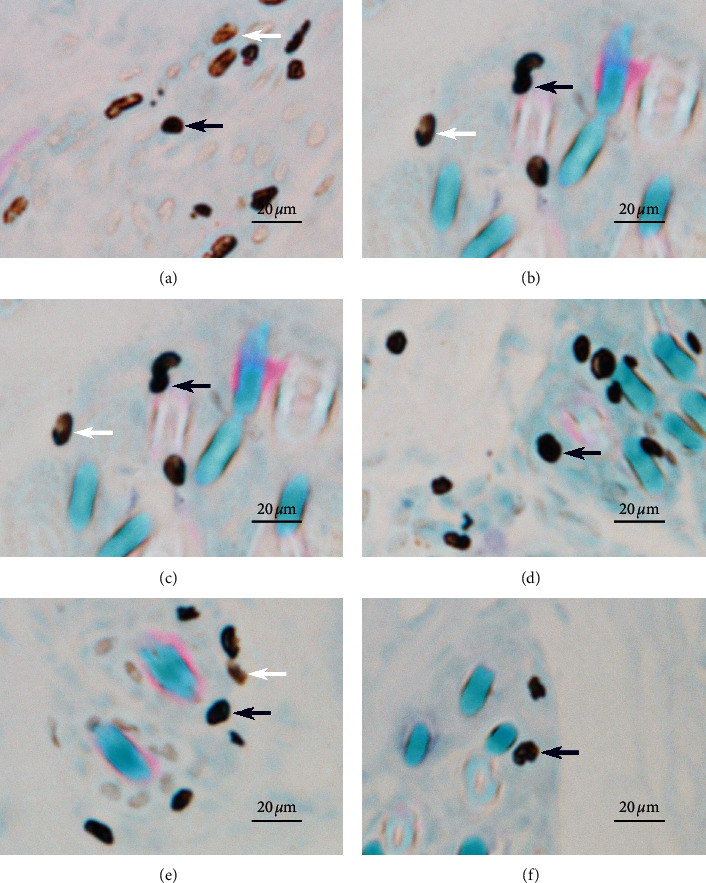
The degranulation of mast cells in acupoints and other points after EA. (a) Degranulation of mast cells in PC6 after EA. (b) Degranulation of mast cells in PC con 1 after EA. (c) Degranulation of mast cells in PC con 2 after EA. (d) Degranulation of mast cells in LU7 after EA. (e) Degranulation of mast cells in LI11 after EA. (f) Degranulation of mast cells in nonacupoint after EA.

**Figure 7:**
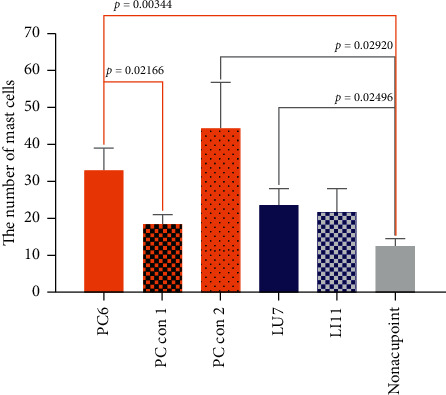
Number of mast cells in rabbit acupoints and control acupoints. The density of mast cells was measured from 6 different rabbit groups under 230 *μ*m × 310 *μ*m scope (*n* = 4∼6). The graph shows groups of rabbits, and the ordinate is the number of mast cells in units of 1.

Many recent studies have shown that mast cells play an important role in the activation of acupoints [25–27]. Tissue comprising acupoints and control points were sectioned and stained (Figure 6). Under a 400× upright dissecting microscope, the diameter of mast cells was approximately 10∼20 *μ*m. Intact mast cells were elliptical or fusiform in shape with clear edges and a dark brown-purple color (cells indicated by blue arrows in Figure 6). Degranulated mast cells were elliptical or fusiform with incomplete and blurred edges or irregularly broken sheets, and the color was light brown (cells indicated by white arrows in Figure 6). Mast cell staining results of both acupoints and control points in EA rabbit groups are shown below. The density of mast cells in the acupoints and control acupoints of rabbits in the EA groups is shown in Figure 7. The average number of mast cells ranged from 12 to 44 in the field of view of 230 *μ*m × 310 *μ*m. The PC con 2 group exhibited a maximum mast cell number, and the nonacupoint group possessed the minimum. Compared to the PC6 group, mast cell numbers in the nonacupoint group and the PC con 1 group were significantly lower. Compared to the nonacupoint group, mast cell numbers in the LU7 group and PC con 2 group were significantly higher.

**Figure 8:**
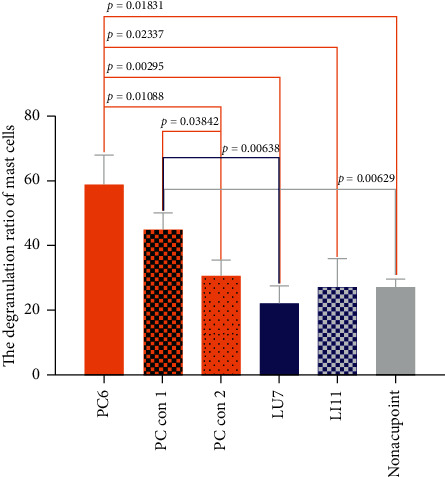
Degranulation ratios of mast cells in different EA groups. The degranulation ratio of mast cells at corresponding acupoints and control acupoints of rabbits was measured in 6 EA groups in a scope of 230 *μ*m × 310 *μ*m (*n* = 4∼6). The abscissa shows the rabbit group, and the ordinate indicates the ratio of degranulated mast cells as a percent.

The degranulation ratio of mast cells in acupoints and control acupoints of different rabbit EA groups is shown in Figure 8. The mast cell degranulation ratio in the rabbit EA groups ranged from 22% to approximately 59%. The degranulated mast cell ratio was the highest in the PC6 group and the lowest in the LU7 group. Except for the PC con 1 group, the degranulation ratio of mast cells in the other groups was significantly lower than that in the PC6 group. The ratio of degranulated mast cells in the PC con 1 group was significantly higher than that in the nonacupoint group, the LU7 group, and the PC con 2 group.

### 3.3. Effect of EA on Adenosine Concentration in Acupoints and Control Points

**Figure 9:**
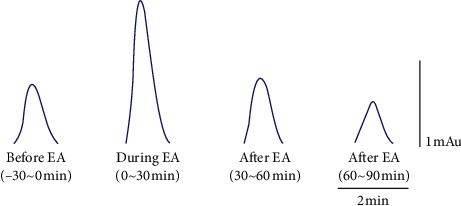
Original HPLC data of samples from a rabbit in the PC6 group. From left to right, samples were collected 30 min before EA at PC 6, after 30 min of EA, 30 min after EA, and from 30 min to 60 min from EA. It can be seen that the adenosine concentration rose during the early EA and fell again after stopping EA.

**Figure 10:**
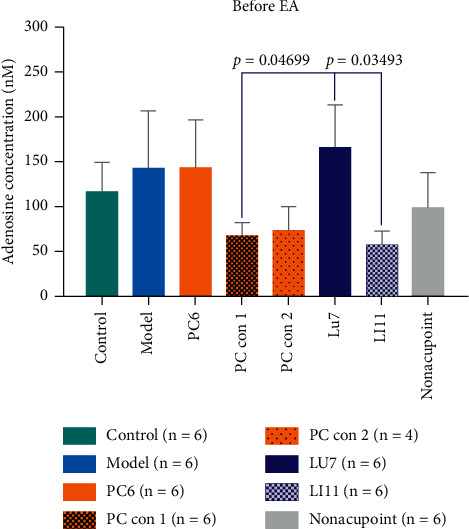
Absolute adenosine concentration in the resting state before EA in different groups. The abscissa shows the groups, and the ordinate indicates the adenosine concentration in units of nM.

Some studies have indicated that adenosine and A1 receptor are involved in the acupuncture effect [28, 29]. To explore whether adenosine participates in the activation of acupoints in acupoint specificity, we used HPLC to measure the adenosine concentration in acupoints and control acupoints. The original HPLC data of samples from a rabbit in the PC6 groups is shown in Figure 9.  Figure 10 shows the absolute adenosine density of acupoints and control acupoints in the resting state. Adenosine samples in the control and model groups were collected from the PC6 acupoint. There was no significant difference between acupoints and control acupoints, except for LU7. Adenosine concentrations in the LU7 group were significantly higher than those in the PC con 1 and LI 11 groups.

**Figure 11:**
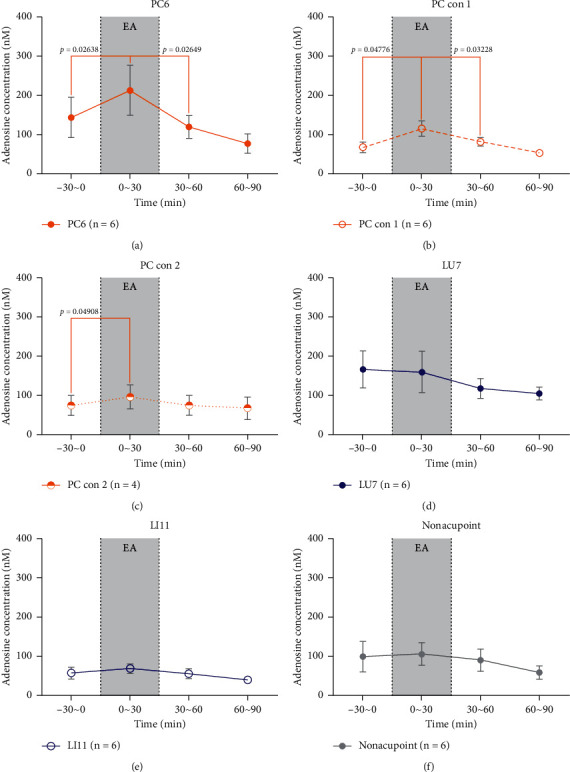
The absolute value line chart of adenosine concentrations in different groups The abscissa shows time in units of min. The ordinate indicates adenosine concentrations in units of nM. From left to right, they are samples collected from 30 min before EA (−30∼0 min), samples collected at the time of EA (the time of EA is 30 min), samples collected from 30 to approximately 60 min after EA, and samples collected from 60 to approximately 90 min after EA. (a) PC6 group. EA caused a short-term increase in absolute adenosine concentration in the local area of PC6, which was significantly different from those in the -30∼0 min and 30∼60 min periods. (b) PC con 1 group. EA caused a short-term increase in absolute adenosine concentration in the local area of PC con 1, which was significant compared to the -30∼0 min and 30∼60 min periods. (c) PC con 2 group. EA caused a short-term increase in absolute adenosine concentration in the local area of PC con 2; while these levels were significantly different compared to samples collected 30 min before EA, there was a difference compared to samples collected after EA. (d) LU7 group. EA did not cause a short-term increase in adenosine concentrations in the local area of LU7. Rather, it caused a decrease, and there were no significant differences compared to the −30∼0 min or 30∼60 min periods. (e) LI11 group. EA caused a short-term increase in adenosine concentration but was not significantly different compared to samples collected 30 min before and after EA. (f) Nonacupoint group. EA caused a short-term increase in adenosine concentration, but this increase was not significant compared to the -30∼0 min or 30∼60 min period.

Figure 11 shows the changes in adenosine concentrations at different acupoints and control acupoints in the rabbits we studied. As shown in Figure 11, only acupoints and control acupoints on the pericardium meridian caused a short-term increase in the adenosine concentration, which had a significant advantage compared with that in the -30∼0 min or 30∼60 min periods. Giving EA stimuli at other body surface points, including other meridians acupoints (LU7, LI11) and nonacupoints, caused no significant differences in adenosine concentration compared with that in the –30∼0 min or 30∼60 min periods. These results suggest that there is a relative specificity in alterations of adenosine concentration at acupoints and control acupoints along the pericardium meridian in response to EA stimulation in rabbits.

## 4. Discussion

Acupoint specificity is one of the cores of traditional Chinese medicine acupuncture theory. In the concept of traditional Chinese medicine, acupoints are specific surface points on the human body that show therapeutic effects after stimulation by acupuncture, massage, or moxibustion. The concept of acupoint specificity includes three aspects. The first is the specificity between acupoints of different meridians [30], which means that each meridian has its unique therapeutic effect, while the acupoints on the same meridian have the same main therapeutic effects. The second is the difference between different acupoints of the same meridian [30]; although the same meridian acupoints have similarities in their main therapeutic effects, there are still differences in the distribution of these acupoints on the human body, and the main therapeutic effects are not entirely the same. The third is the specificity between acupoints and nonacupoints [30]. Nonacupoints, which refer to body surface points not on the traditional meridian track line or deviate from acupoints, have a further distinct difference compared with acupoints. From a generalized perspective, acupoint specificity can also indicate that an acupoint has a relative specificity in morphology [31–33], biophysical characteristics [34, 35], pathological response [36, 37], or therapeutic effect [16, 19, 26] compared with other acupoints and nonacupoints.

Clinical practice suggests that acupoint specificity plays an important role in guiding clinical treatment [7, 8, 38]. Using the improved rabbit acute bradycardia model, a suitable animal model for the human bradycardia disease [26], we found that stimulating PC6 (the pericardium meridian, a clinical indicator of angina [39, 40], arrhythmia [41], and gastritis [42]) with EA induced a significant increase in heart rate during bradycardia induced by acute myocardial ischemia in rabbits, and the recovery of relative heart rate was significantly higher than that in the LU7 (the lung meridian, a clinical indicator of cough [43] and headache [44]) and LI11 (the large intestine meridian, a clinical indicator of arm pain [45] and hypertension [46]) groups (Figures 4 and 5). This indicates that the effect of acupuncture cannot be obtained by applying EA stimulation on any random point on the body. As mentioned, in traditional Chinese medicine theory, different acupoints and meridians have different outcome effects, but acupoints on the same meridian may have similar effects. In this study, acupoints and control acupoints on the pericardium meridian show a relative specificity in the response of adenosine to acupoints on other meridians and nonacupoints. However, with respect to the acupuncture effect, we found that only applying EA stimulation at PC6 exerted significant recovery on heart rate. Although the relative heart rate recoveries of control acupoints on the pericardium meridian and acupoints on other meridians were higher than those in the model group, these differences were not statistically significant.

Besides EA, the operation of embedding a linear microdialysis probe can also cause an increase in adenosine concentration. That is the reason we had to perform EA stimulation one hour after finishing planting the linear microdialysis probe, aiming to reduce the adenosine concentration at the acupoints to a reasonable level. We noticed that the adenosine concentration at LU7 before acupuncture, while showing a descending trend, was significantly higher than that of PC con 1 and LI11 (Figure 10). It may be because of the different directions between the buried linear microdialysis probe and the acupuncture needle to stimulate LU7 during EA. The direction of the buried linear microdialysis probe embedded under the skin of LU7 is perpendicular to the plane of the radius and ulna. Nevertheless, the acupuncture needle stimulating LU7, which forms an acute angle with the line segment between LU7 and radial styloid process, is located on the plane of the radius and the ulna. The LU7 adenosine concentration result may suggest that stimulating LU7 in the direction perpendicular to the radius may be more effective.

**Figure 12:**
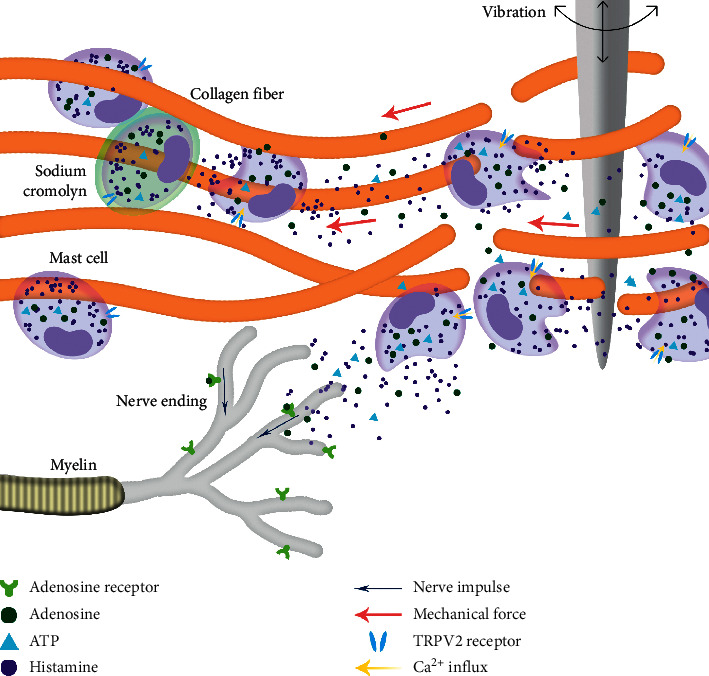
The mechanism of acupoint activation for specific acupoint effects. When the needle was inserted into the acupoint, it could induce contraction-relaxation of the skeletal skin muscle, thereby causing local collagen fiber entanglement and deformation. The resulting mechanical force was transmitted to the membrane of the mast cell, inducing the TRPV2 channel on the mast cell membrane to open. It then caused a large amount of calcium influx, activated mast cells, and released intracellular mediators such as histamine, ATP, and adenosine. Adenosine from intracellular and ATP hydrolysis bound to the adenosine A1 receptor on nerve endings to produce a nerve impulse and trigger acupuncture effects. Sodium cromolyn, a mast cell membrane stabilizer, blocks the acupuncture effect by inhibiting the degranulation of mast cells.

To explore differences in the mechanism of acupoint activation, we sectioned tissues from three acupoints and observed the mast cells. The results showed that the density of mast cells in the acupoints on pericardium and lung meridians was higher than that in the nonacupoint, which is consistent with the observation of human anatomy that the densities of cutaneous mast cells are highly relative to the distribution of acupoints (Figure 7) [47]. Differences also could be found in the ratio of degranulated mast cells. The ratio of degranulated mast cells in PC6 was significantly higher than that in LU7 and LI11 (Figure 8). Previous studies have demonstrated that the ratio of degranulated mast cells is closely related to acupuncture's effect [26, 27, 48]. In this work, the acupuncture effect of PC6 was significant, and the ratio of degranulated mast cells in PC6 was also significantly higher than that in the other two acupoints. This suggests that heart rate elevation may be associated with the activation of mast cells. Similarly, the penetration of a needle into the muscle layer under PC6 and electrical stimulation on the needle significantly increases adenosine concentrations in tissue fluid of the superficial fascia under PC6. However, even with the same current stimulation intensity, this phenomenon was not observed in the other two acupoints (Figure 11). When the needle was inserted into the acupoint, the current caused the needle to vibrate, and the mechanical stimulation induced by the needle caused the collagen fibers to be mechanically deformed [49]. Collagen fibers play a role in transmitting mechanical force [50]. Although the goal of mechanical force transferred by collagen fibers is still unclear, there is evidence that the TRPV2 channel on the surface of the mast cell membrane opens when subjected to mechanical stimulation. This behavior causes a large amount of calcium influx, which activates mast cells and releases certain intracellular mediators [51]. Acupuncture can cause a significant increase in adenosine concentration in interstitial fluid [29]. The adenosine in tissue fluid activates the adenosine A1 receptor on nerve endings to produce the acupuncture signals [28]. Adenosine in tissue fluid may be derived from activated mast cells [52]. One study has shown that injection of sodium cromolyn—a kind of mast cell membrane stabilizer—in acupoints inhibits an increase of adenosine [52]. In addition, mast cell degranulation results in increased extracellular ATP levels [53, 54], and adenosine is a hydrolysate of ATP. Therefore, we have reason to believe that elevated adenosine in the tissue fluid during EA is partly derived from degranulated mast cells. Combined with previous studies about the acupuncture mechanism and the results we report, we present a diagrammatic sketch to show the mechanism of acupoint activation for specific acupoint effects (Figure 12).

Stimulating one or several specific acupoints in a nonspecific way achieves various regulating effects of acupuncture on different diseases. However, the existence of acupoint specificity is still controversial [8, 19, 20]. After observing the contrast of regulations on heart rate induced by acupuncture application on various acupoints and control points based on the rabbit acute bradycardia model, we tried for the first time to explain the physiological mechanism behind it from the view of acupoint activation and thus put forward the hypothesis that differences based on mast cell degranulation and adenosine release may be one potential physiological basis of acupoint specificity. The effect of applying acupuncture at PC6 on the heart rate regulation of acute bradycardia also provides a new perspective for clinical exploration of the treatment of related diseases and contributes a reference for selecting acupoints. Limited by acupuncturists' actual operation levels, not every acupuncture practice can achieve the expected therapeutic effect. However, we can provide targeted suggestions to improve acupuncture efficiency based on probing the acupoint activation mechanism.

It should be noted that acupoint specificity is a comprehensive proposition. In this study, we only observed PC6 and two control points along the pericardium meridian, LU7 on the lung meridian, LI11 on the large intestine meridian, and the nonpoint beside PC6. Limited samples cannot draw a general and universal conclusion, and the mystery of acupoint specificity urgently needs more scientific research to unveil.

## 5. Conclusions

In summary, our current data led us to speculate that the biological basis of relative acupoint specificity may be based on mast cell degranulation. Acupuncture induces mast cell degranulation to release adenosine, and adenosine activates the A1 receptor on sensory nerves to induce the acupuncture effect in regulating heart rate (Figure 4). Compared to other acupoints, the biological mechanism of the advantage of PC6 in modulating heartbeat is based on differences in the mast cell degranulation ratio and adenosine concentrations in the tissue fluid. Similarly, the difference between PC6, PC con 1, and PC con 2 illustrates that differences between PC6 and nonacupoints in heartbeat modulation are also based on the same basic biological mechanism (Figures 5, 8, and 11). Moreover, we hypothesize that, in an acute myocardial ischemia rabbit model, the reason for the advantage of PC6 in the acupuncture effects compared with other meridian acupoints and control acupoints is the differences in adenosine concentrations in tissue fluid following acupuncture.

## Data Availability

The datasets used and/or analyzed during the current study are available from the corresponding author on reasonable request.

## Abbreviations

EA: Electroacupuncture
Con: Control
HPLC: High-performance liquid chromatography
ECG: Electrocardiograph.
